# Perceptions on technology for volunteer respite care for bedridden elders in Chile

**DOI:** 10.1080/17482631.2017.1422663

**Published:** 2018-01-16

**Authors:** Esmeralda Abarca, Solange Campos, Valeria Herskovic, Carolina Fuentes

**Affiliations:** ^a^ School of Nursing, Pontificia Universidad Católica de Chile, Santiago, Chile; ^b^ Department of Computer Science, Pontificia Universidad Católica de Chile Carolina Fuentes, School of Computer Science, University of Nottingham, Nottingham, United Kingdom; ^c^ School of Computer Science, University of Nottingham, Nottingham, United Kingdom

**Keywords:** Caregiving, IT, respite, volunteer, qualitative research

## Abstract

**Purpose:** Informal caregivers of bedridden elders need a respite. One form of obtaining a respite is through volunteers who are contacted by means of information and communication technology (ICT). **Method:** A qualitative study was carried out in a low-income district in Santiago, Chile, to learn about how caregivers of bedridden elders perceive the possibility of using ICT to access this respite. In-depth interviews were carried out and transcribed verbatim, then analysed using open coding. **Results:** The results reveal that caregivers are willing to receive a volunteer in their home and use ICT to communicate with them, although a discrepancy exists between the use of devices connected to the Internet and feature phones. **Conclusion:** This study concludes that informal caregivers of bedridden elders have a favourable disposition towards accessing a respite system by means of ICT based on a peer-to-peer economy.

## Introduction

The global population is ageing. It is estimated that by the year 2050, the proportion of senior citizens will exceed 30% in several countries, including Chile (World Health Organization, 2015). While senior citizens in general are independent and capable of directing their own lives, a significant number are expected to develop chronic illnesses that diminish their functioning and increase their dependence on caregivers to carry out basic activities of daily living (BADLs) as well as instrumental activities of daily living (IADLs) (Chatterji, Byles, Cutler, Seeman, & Verdes, 2015). The percentage of elders over 75 years of age that depend on caregivers for at least one BADL varies between 17% in Switzerland and up to 40% in Mexico or the Russian Federation (World Health Organization, 2015). In Chile, according to a national study carried out in 2009, 12.4% of senior citizens aged 60 were severely dependent on a caregiver (bedridden people and/or those with any stage of dementia, among other ailments) (Servicio Nacional del Adulto Mayor, 2010).

In general, dependent senior citizens are cared for by family members or those close to them who act as informal caregivers, with the objective of satisfying the BADLs and IADLs. The provision of care can last a long time and encompass the physical, psychological, social, spiritual and existential areas of a person (Servicio Nacional del Adulto Mayor, 2010). The demand that is required of a caregiver to satisfy all of the care-recipient’s needs puts the caregiver at risk of developing emotional stress, physical ailments, work abandonment and family conflict, among other issues (AARP, 2015a; Riera et al., 2015; Toms, Quinn, Anderson, & Clare, 2015). As a result, the weight of the caregiver’s responsibility takes shape, and has been defined as a dynamic process resulting from negative emotions and experiences that are characterized by negative effects at the physical, emotional and psychological level, (M.-C. Chen, Chen, & Chu, 2014; Del Río-Lozano, García-Calvente, Marcos-Marcos, Entrena-Durán, & Maroto-Navarro, 2013; Werner, Mendez-Luck, Kennedy, & Wallace, 2008; Yen, Huang, Ma, Lee, & Lee, 2009). The caregiver’s respite, or break, is the most common of all of the caregivers’ needs (AARP, N. A. for C. (NAC), 2015a). This respite is understood as planned or sudden care for a person with special needs with the aim of temporarily relieving this responsibility from the family caregiver (American Psychological Association, 2017). The need for this respite favours the establishment and maintenance of a social network between community services and the caregiver-recipient binomial, which would contribute to the reduction of social isolation and the improvement of the quality of life for those involved (Evans, 2013a, 2013b). The respite can be accessed by replacing the caregiver with a well-qualified volunteer, ideally trained and with adequate geographical access (AARP, 2015b; Jegermalm & Grassman, 2009). A conceptual model of respite encompasses three attributes: Partnership (relationship between care recipient, caregiver, and respite provider), Service (what the respite provider offers) and Respite (what the caregiver and care recipient obtain from the respite) (Evans, 2013a).

The coordination and the link between community volunteers and the informal caregivers are necessary in order to provide them with a respite. This coordination can be carried out through the use of ICT by means of a peer-to-peer economy (Ellison, Steinfield, & Lampe, 2007; Voida, Harmon, & Al-Ani, 2012). A peer-to-peer economy refers to diverse services provided by individuals or organized communities, directed towards people that require such services. These economies have three essential characteristics: (1) they are based on computational technologies in which the power and production value are distributed between users, (2) there is an efficient use of goods, work, materials and knowledge or any other asset on behalf of the user, and (3) it is associated with the local area or a community, whose members live relatively close to one another (Bellotti et al., 2015; Bellotti, Cambridge, & Hoy, 2014; Botsman, 2013).

The benefits provided by platforms based on peer-to-peer support within communities of senior citizens are varied. Some examples include the strengthening of communication within the community, a rise in social capital, improved perception and delivery of support, and an increase in peoples’ wellbeing, in particular senior citizens (Moser, Krischkowsky, Neureiter, & Tscheligi, 2015).

In order to use computational technologies and take advantage of the peer-to-peer economy, people must have certain digital skills. Digital skills comprise the critical use and confidence in the use of ICT for working, learning, communicating and amusement, as well as the ability to participate and communicate in social networks through the Internet (DG CONNECT F4, 2014). In the health sector, for example, digital skills can benefit users by means of linking people to resources and support systems; and in the cultural and civil realm through enabling access to information, freedom of expression and interaction, among other benefits (Ala-Mutka, 2011). A 2012 study revealed that the percentage of the general population that was not digitally competent was 6% in Sweden, 23% in the U.S.A. and 50% in Romania. The same study also showed that in Sweden, the U.S.A. and Slovenia, 11%, 38% and 50%, respectively of people with low levels of education or low incomes were not digitally competent (DG CONNECT F4, 2014). In Chile, the results from the Survey of Adult Skills (OECD, 2016) made in 2016 by the Organization for Economic Co-operation and Development (OECD) revealed that 69% of adults between 55 and 65 years old in Chile do not have experience or fail in the use of technology. This rate decreases to 9% among youngest population (16–25 years old) (MINEDUC, 2016). In light of the critical need to support informal caregivers of bedridden elders, as well as the technological developments that could help generate a peer-to-peer economy, this study aims to learn how caregivers of bedridden elders perceive the acceptability of ICTs that enable them to access a respite.

## Methods

The study was exploratory, descriptive and comprehensive, using a qualitative methodology that involved researching the perceptions of caregivers of bedridden elders regarding the acceptability of ICTs that allow them to access a respite.

### Participants

The study involved the participation of informal caregivers related to bedridden elders. Each participant was registered in the primary level of a healthcare program. The criteria for participation included: being the primary caregiver of an elder for a minimum period of 6 weeks, over age 18 and literate. The criteria for exclusion included having a disability or functional limitation such as deafness or blindness. Twelve interviews were conducted, although two were excluded (one did not fulfil the inclusion criteria, and one caregiver was too emotionally distraught to participate in the research). The participants included 10 informal caregivers: nine women and one man. The average age was 53.7 years (38–77), and the average education was 10.4 years (8–17). Of the women, four were housewives, two were assistants in the fields of nursing and education, one a metalsmith, and two were unemployed. The male participant was a public transport driver. The period of caregiving time was between one and 20 years (an average of 8.3 years). Combined, they cared for 11 senior citizens total (one person cared for two elders), of which two were men and nine were women between the ages of 68 and 88 (80 was the average). All of the elders had a maximum degree of dependency on the caregivers (evaluated under the Lawton-Brody scale and Barthel index) and were bedridden. Six people cared for their mothers, two people looked after their fathers, and one cared for her husband, another her father in law, and one person cared for both parents. All of them lived with the elder they cared for.

Regarding the digital skills of the caregivers, this was measured using the EU wide indicators of Digital Competency methodology, which measures four areas of abilities: information, communication, content creation and problem solving. The indicators present three skill levels: “none,” “low,” “basic” and “above basic” (DG CONNECT F4, 2014). The results indicate that the majority was not digitally competent and only one participant had above basic skills. Even though all of the participants owned and used a cellphone, only four of them had smartphones. Six caregivers had Internet connection at their homes.

The participants were contacted through a list of caregivers provided by the primary care healthcare centre. Then, they were invited to participate in the study by means of a phone call from the researchers. A time was arranged to meet with those that accepted the invitation. Finally, participants who accepted with informed consent in the investigation participated. A compensation of 10,000 Chilean pesos ($15 USD) was given to those participants that accepted.

### Data collection

In-depth, semi-structured interviews were carried out by one researcher in the participants’ homes, upon the request of the participants. The questions used to guide the interviews are provided in Appendix A. Afterwards, the interviews were recorded and transcribed verbatim.

### Data analysis

The researchers developed a qualitative descriptive analysis of the data produced from the interviews. Specifically, they applied an open code analysis based on the grounded theory methodology. This analysis enabled the generation of codes that were grouped into subcategories which subsequently shaped the main categories (Charmaz, 2014; Corbin & Strauss, 2015).

To ensure the methodological rigour in the quality of the results, the researchers applied the triangulation strategy (Corbin & Strauss, 2015). Two researchers participated in this stage of the investigation, specifically in the qualitative analysis of the data. For each interview, the researchers analysed it independently and then met to discuss discrepancies until a consensus was reached and the researchers reached an intersubjective agreement on the categories produced. In addition, the two final interviews confirmed the saturation of the main categories. To strengthen the credibility of the study, the results were presented to two of the people interviewed.

### Ethical considerations

The project was approved by the Scientific Ethical Committee of the Pontifical Catholic University of Chile (Project Number 15–334). Participants agreed to participate with informed consent.

## Results

**Figure 1.**
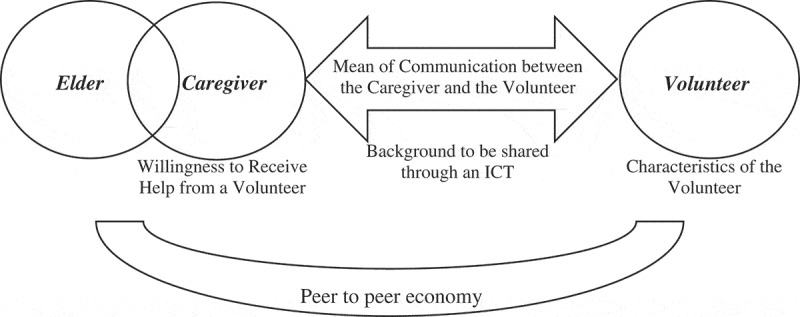
Descriptive analysis: a diagram of the main categories.

Following the descriptive analysis of the 10 interviews with the caregivers of bedridden elders, four main categories emerged: willingness to receive help from a volunteer; characteristics of the volunteer; means of communication between the caregiver and the volunteer; and background information to share by means of information technology. These categories will be explained in more detail in the following pages and are shown in Figure 1.

### Willingness to receive help from a volunteer

The caregivers explicitly stated their willingness to receive help in their home, referring to the need to work without leaving the bedridden person without the required care; the lack of support networks for caregivers in the community to facilitate a respite; and a lack of time to carry out personal and domestic tasks, such as running errands, shopping, going to the doctor and having leisure time.

*“It would be a huge help, a huge help for many people. Sometimes I feel very stressed out from having so much work, but I know that there are other people that do not have anyone, no one to look at, nor to help you; there are many people that really need this help.” (C8)*

Moreover, the caregivers laid out certain obstacles to receiving help. First, the bedridden person would be accustomed to being cared for by the habitual caregiver, and therefore they could not be replaced by a stranger. In addition, the caregiver would be concerned about the elder’s state of health in their absence, and fear they would not be present if a crisis or even death occurred. Therefore, if the caregivers receive voluntary help it would be requested at specific times during the day or for a limited period. Other difficulties include the fact that the caregiver is not the home owner and therefore could not decide whether a volunteer could enter the home and assist the elder, or whether the volunteer had the minimum physical conditions to properly carry out the necessary work.

*“The caregiver becomes emotionally dependent on the sick elder, hoping that the person dies in a moment when they are there. But if you leave even for just a day and you arrive, or in the middle of the day they call you and tell you they had a heart attack and passed on… you could not imagine the feeling.” (C2)*

### Characteristics of the volunteer

Each volunteer must have certain characteristics that correspond to special capacities, skills, attitudes and certain requirements related to age, experience and membership of an institution. The volunteer must be empathetic, know how to handle the elder during mood swings, guide them in different situations and according to their pathology, possess knowledge of basic care for an elder and be able to listen and establish a conversation with this person. Additionally, they must be in suitable physical condition to be able to move an elder lying in bed or sitting in a chair and ensure they do not fall. The person must also have good hygiene and tolerance for tasks such as changing diapers. Finally, the caregivers mention that the volunteer could be a student from the health sector that already has some knowledge of basic care.

*“I would find a volunteer for my father that knew everything about his illness… that maybe had the ability to listen… to link up his story when he feels like talking, and someone who has the capacity and ability to calm him down…” (C2)*

#### Guidelines for the volunteer

The volunteer could be a young person as long as they have experience in caring for elders. In addition, they could be younger than the habitual caregiver considering they may be more agile, or they could be an experienced middle-aged person who is able to listen, chat, give advice and humour the elder, which would generate trust.

*“Really, really young, no… But if the person has worked in or […] has studied this area, such as nursing, they know how to treat them because they have worked with adults…” (C1)*

The volunteer must have a vocation, be tolerant, honest, calm, responsible and perseverant, have good will, and have the skills to satisfy the elder’s basic activities of daily living.

*“The person must have twice the vocation, because they are working with strangers…” (C1)*

One notable characteristic of the volunteer desired by the caregiver is membership of an institution, which would generate trust in the caregiver, guarantee that the volunteer is responsible and has the capacities necessary to assist a bedridden elder. The institution would be responsible for seeking out, contacting, obtaining background information on and selecting the volunteers. These volunteers would then provide the habitual caregivers with a respite and meet the conditions previously stipulated by the habitual caregiver.

*“People that come and that you know are responsible can stay, especially if they have worked with other people… (this way you can) trust that this person is responsible and that they will be able to accompany my husband…” (C5).*

*“…that they came from a place, like an organization. This generates trust […] and this way I have a number I can call and really get to know what this person is like, how they work in this place, how they perform their duties or if they had the experience, maybe with another bedridden person…” (C9).*

#### Requirements for receiving a volunteer

The caregiver must have general knowledge of the volunteer prior to beginning to job, such as their residence, telephone number and whether the person is trustworthy. Additionally, the caregiver and the volunteer must meet beforehand in order to speak face-to-face and establish agreements, and also discuss suggestions or comments from people that previously knew the volunteer. If the volunteer cannot assist an elder more than once, the caregiver is required to meet the new volunteer that will be assisting the elder. Crucially, the caregiver should have a basic trust in the volunteer based on the fact that this person is associated with a volunteer initiative supported by an institution. A caregiver can request that the volunteer remain the same and that, if replaced, the new volunteer possess similar characteristics.

“To learn more about the person face-to-face, rather than by means of a computer… I would have to meet the person before they came here, meet them first, talk with her in order to learn what this person is really like, because seeing this person on a computer is not the same thing…” *(C10)*

Regarding the schedule, help would be needed in the morning or in the afternoon, from 9:00am to 4:00pm, according to the caregiver’s routine and activities. The number of hours per day may vary from two to 6 hours. Additionally, the number of times per week required could range from two to three times, or every other day. A caregiver would also request assistance one weekend a year to take a vacation.

“…so that this caregiver can leave for ‘a few hours,’ which means two or three hours…” *(C2)*

#### The type of help needed from a volunteer

The care given to the adult on behalf of the volunteer should include certain characteristics, among them considering the activities the elder enjoys, adapting to the routine of the elder and being dedicated exclusively to him or her. Additionally, the caregiver would expect the volunteer to fulfil certain objectives, such as keeping the elder active and avoiding falls. Additionally, the care given by the volunteer should not be rushed, and keep to the times and routines established by the elder. The care should also be carried out with affection and respect.

*“… the volunteer should be patient and not mistreat my father because in the end it will worsen his state even more… so they must be patient and not mistreat him, and be loving,” (C10)*

The volunteer must accompany the elder, and actively listen and chat with them. Communication with the elder must be carried out in the appropriate volume and at an audible level, and be paused as often as needed. The elder’s recreational activities would require the volunteer to read a book, play the music desired by the elder, or turn on their favourite show on the television.

*“…listen and talk with her… they could be her companion and listen to her…” (C4)*

According to the informal caregivers of bedridden elders, the volunteers must also assist the elder in areas such as hygiene, skincare, eliminating urine and faeces, transport and movement, as well as getting out of bed. The act of changing the elder’s diapers must depend on whether or not the elder wishes to do so with the help of the volunteer, even if it is necessary to do, without the presence of the informal caregiver. Instrumental activities include the preparation and delivery of food, and the administration of medication according to the schedule.

*“…comb her hair. She always wants you paint her nails, cut and file them…” (C1)*

*“…if a person came to help us, it would be to move her.” (C3)*

*“give her medication […] and give her lunch on time with a little salad.” (C5)*

### Means of communication between the caregiver and the volunteer

This category refers to the means of communication the caregiver prefers to use to connect with a potential volunteer.

#### Using a feature phone

Some caregivers feel that using a common, feature phone (without the advanced functionalities of smartphones) would be the most comfortable way for them to contact a potential volunteer and that other means such as Facebook would not ensure they would be able get to know the potential volunteer adequately. This device would allow them to communicate when necessary, would be easy to use, and would connect to phone numbers they use frequently.

*“I don’t like profiles… the idea of putting up a picture like on Facebook. This allows you to get to know the person physically more than anything, but you can’t see what the person really is like.” (C4, female caregiver, 48 years old, and with no digital skills)*

*“So if I meet this person it would be through a cell phone like this, a ‘normal’ cell phone as I call it,” (shows her cell phone, an Alcatel flip phone), normal meaning that it allows you to make calls and that’s it, cell phones like those for the elderly with the numbers inside and if they want to make a call, they press a button and call. For me this is more practical…” (C6, female caregiver, 52 years old and with no digital skills)*

#### Using a device with internet connection

Some caregivers consider the use of a device that is connected to the Internet useful, such as a computer or a smartphone, in order to easily and quickly access voluntary help. One caregiver suggested that the creation of a group of informal caregivers on WhatsApp would be useful to support one another.

*“Look at this app. It’s meant for communication between me and this person, and nobody else will read it.” (C4, female caregiver, 48 years old, without any digital skills)*

*“Creating a group, for example, a group on WhatsApp of all the (caregivers) that they are going to visit and from there, one can help by exchanging ideas or something like that, and say ‘look at this [volunteer], I like them more than the other one…’ it’s like creating a network and between us, supporting one another.” (C9, female caregiver, 50 years, with low digital skills)*

### Background to be shared through an ICT

The caregivers suggest that, among the means of communication between the caregiver and volunteer, background information on the elder and the volunteers could be available, which would ultimately enable the volunteer to do a better job. For the elder, the background information would include a description of their character, their condition, daily routine, diet, their need of companionship, and the extent of their movement. In turn, the information for the volunteer would include their age, prior experience caring for an elder, knowledge of disease processes and care, and the reason why they are volunteering.

*“And explain the routine in the case that she cannot do something or she does not like it. Explain to her how I do this with him, the schedule and his medication, the time he gets up, the time he eats his meals and what exactly he eats.” (C5, female caregiver, 77 years old, without any digital skills)*

*“That this is a person of a certain age, that they are helping the community, that maybe they are someone who needs help or that wants to do this to change their usual routine… Knowing this is important…” (C4, female caregiver, 48 years old, without any digital skills)*

## Discussion

We have described the requirements for voluntary respite care. The results show that caregivers are willing to receive this help. The reasons that support this claim are related to the need to carry out other activities which they are unable to carry out while caring for an elder. This is also supported by evidence in the literature that explains that caregivers would devote their free time to carrying out household chores, medical assistance, rest, recreational activities and work (Greenwood, Habibi, & Mackenzie, 2012; Mastel-Smith & Stanley-Hermanns, 2012; Moule et al., 2014; Stirling, Dwan, & Mckenzie, 2014).

The three-attribute conceptual model proposed by Evans (Evans, 2013a) provides a useful frame for the results obtained in this study. The *partnership* between caregiver, care recipient, and respite provider should be the central component of respite service, although caregiver and care recipient may have different outlooks and benefit in different ways from respite care. The present study found that the caregivers would accept volunteer respite care as long as the dependent person felt or appeared comfortable and agreed to letting another person care for him or her, which has also been reported by other authors (Greenwood et al., 2012; Neville, Beattie, Fielding, & MacAndrew, 2015; Stirling et al., 2014) and highlights the importance of the relationship between all three stakeholders.

Although caregivers are willing to access respite care, they lay out obstacles similar to those stipulated in the literature, such as the need to constantly supervise the dependent person due to reasons such as concern for the elder’s safety and a heightened sense of responsibility and guilt that makes it difficult to leave the elder alone (Greenwood et al., 2012; Moule et al., 2014). The present study further examines these obstacles to a successful partnership, also relating the desirable traits of the volunteer, where the caregivers consider that the person providing the care, whether paid or voluntary, provide emotional support and have knowledge on caring for elders; at the same time, it is of the highest importance that the person have prior experience in this work (Greenwood et al., 2012; Neville et al., 2015; Stirling et al., 2014). Another desirable characteristic of the volunteer, reported by the caregivers, is that they should belong to a volunteer service institution. Similar results were found in the UK, where informal caregivers of elders trusted in the good service provided by paid and voluntary caregivers due to the fact that they were recommended by a government agency (Greenwood et al., 2012). Several studies report that the informal caregiver’s trust is partially based on its trust in the institution that provides the respite service, as well as its trust in the person providing the care. This is shown in the feedback of the person receiving the care on the care received (Greenwood et al., 2012; Stirling et al., 2014). Another result shows that, in order for the caregivers to receive a volunteer in their home, they would require meeting the volunteer in person beforehand, and that the selected caregiver commit to regular visits. Moreover, various caregivers that participated in the Moule et al. (2014) study had a positive experience when accessing the respite by means of a face-to-face interview, preferring personal contact over a telephone call. Along the same line, the informal caregivers of dependent people would enjoy the opportunity to speak and simply be heard by the respite care provider, whether paid or not (Greenwood et al., 2012; Neville et al., 2015). Regarding the regularity of the service, the caregivers recommend that the respite be carried out on a regular basis according to the schedule they require. A characteristic of the respite is described in the literature as an element that will allow the caregivers to plan their free time in advance (Greenwood et al., 2012), bringing relief to the caregiver. Additionally, Evans’ conceptual model of respite incorporates duration and flexibility as elements of the respite service; the former considers the time of the respite in hours, days or longer-term stay, while the latter considers the degree of adaptation to the service in terms of the caregiver’s selection of hours and length of time (Evans, 2013a). In effect, the lack of flexibility would be a barrier to accessing a respite (Jeon, Chenoweth, & McIntosh, 2007; Neville et al., 2015).

Regarding the *service* attribute (Evans, 2013a), the type of help expected of the volunteer in this study is focused on the *assistance* dimension, comprising the provision of companionship, recreation for the elder, help with the BADLs and IADLs in a respectful and loving manner, with exclusive dedication to the dependent person. These characteristics are similar to those referred to by informal caregivers of dependent people, who consider that the treatment of the elder should be “lovely” and “kind” (Greenwood et al., 2012), and that the dependent person should receive quality care centred around the care-recipient’s daily routines and needs, as well as exclusive dedication that allows them to remain active and happy (Stirling et al., 2014). The caregivers of people with dementia also note the importance of delivering care that is centred around the care-recipient (Neville et al., 2015; Phillipson & Jones, 2011). In fact, in Greenwood’s (2012) study, the caregivers of elders considered discontinuing the respite service if the replacement did not attend to the needs of the elder, did not adapt to their routine and was not happy. Among the recreational activities that the caregivers expect the volunteer service to carry out is reading, watching television, listening and chatting. This is part of the outcome of volunteer caregiving, or the *respite* attribute (Evans, 2013a), and clearly coincides with the findings of several studies with the aim of ensuring the dependent elder has a pleasant experience by means of social interaction with the volunteer (Greenwood et al., 2012; Neville et al., 2015; Stirling et al., 2014). Precisely, the stimulation resulting from socialization with the caregiver is the most notable benefit of the respite mentioned by caregivers (Evans, 2013a, 2013b; Greenwood et al., 2012; Laverty, Arber, & Faithfull, 2016; Moule et al., 2014; Neville et al., 2015).

The main result of this investigation is that the caregivers consider that the use of ICT to access and continue the respite would be beneficial. However, a discrepancy exists as to the type of technology used. This discrepancy in the use of technology could be associated with the age of the interviewee, seeing that an inverse relationship exists between age and the user’s operation level of the Internet and other digital skills (Feufel & Stahl, 2012; Van Deursen & Van Dijk, 2009). However, other factors do influence access and use of technologies and the Internet. For example, the higher socioeconomic level, the greater number of opportunities one has to acquire the latest technology (Choi, 2011; Choi & DiNitto, 2013; Jensen, King, Davis, & Guntzviller, 2010). Attitude is another factor associated with Internet use, where even senior citizens’ use of technology is mediated by the style with which they approach this technology. A more active confrontation or a proactive stance when faced with challenges would increase the probability of using a computer (Czaja & Lee, 2007; Werner, Carlson, Jordan-Marsh, & Clark, 2011). Additionally, an ICT design that does not adapt to the needs of the community is another factor that could affect its use (Czaja & Lee, 2007). Furthermore, Internet usage would enable the informal caregiver to access sufficient information on self-care (Marziali & Donahue, 2006; Petrovic, 2013), and locate respite care service providers in their neighbourhood without having to leave their home (Mastel-Smith & Stanley-Hermanns, 2012; Petrovic, 2013).

Another result relates to the information on the elder and the background information on the volunteer, both of which the informal caregiver would share through use of ICT. This information would be minimal and essential, such as the name, age, state of health and daily routine of the care-recipient, and the name, age, job and past experience caring for elders for the volunteer. This would facilitate the caregiver’s access to a volunteer by means of ICT. It has been reported within the literature that delivering information to others would allow them to generate trust, understanding and intimacy in interpersonal relationships (Varnali & Toker, 2015). In turn, certain privacy rules should be established to uphold the boundaries that protect intimate information (Kezer, Sevi, Cemalcilar, & Baruh, 2016; Petronio, 2002). Various studies show that the reason for sharing information via social networks differs based on age. Senior citizens worry more about privacy and tend to protect it by not sharing information, while young adults tend to reveal information and use Facebook’s privacy measures (Chang, Choi, Bazarova, & Löckenhoff, 2015; Steijn, 2014; Varnali & Toker, 2015). This information should be considered when designing a strategy or ICT that connects caregivers with volunteers, in order to respectfully and effectively safeguard a person’s privacy.

Motivations to participate in a peer economy system are usually mismatched between providers and users: providers are generally more altruistic, while users are trying to satisfy their needs (Bellotti et al., 2015). This study focuses on the users of a peer economy system based on volunteer caregivers, in which this same mismatch is present between the altruistic providers (volunteers) and the users (caregivers and care recipients). Considering the results of this study, one can deduce that the caregiver-elder binomial would benefit from a volunteer service based on a peer-to-peer economy (Bellotti et al., 2015, 2014; Botsman, 2013). Additionally, considering that one of the components of a peer-to-peer economy is the use of technological support, the caregivers that participated in this study expressed that they would be willing to use devices that connect to the Internet, such as a Smartphone, computer or other device. A second component of the peer-to-peer economy is the efficient use of goods, work and knowledge, which based on the results of this study could be reflected in the value of the volunteer’s experience, as well as the social interaction of the dependent person. The third component centres on community belonging, which is reflected in this study through the participants’ affiliation to a local healthcare support program that groups them based on their location. Caregivers would be able to consider volunteers that live in this area as potential replacements.

Furthermore, the literature shows diverse initiatives in which ICTs are used as a means by which caregivers of patients with cancer and Alzheimer connect with community services to obtain information, education, social support and respite. Among the benefits for the caregiver is the development of interpersonal relationships, a reduction in social isolation, and an increase in wellbeing and/or personal satisfaction. These benefits will ultimately affect the quality of life for the caregiver as well as that of the care-recipient (Bellotti et al., 2015; 2014; Y. Chen, Ngo, & Young, 2013; Tixier & Lewkowicz, 2015).

## Conclusion

This study has demonstrated that caregivers of elders in a state of severe dependence find it feasible to access volunteer respite care for their loved one through the use of ICT. The ICTs mentioned include the telephone or a device that connects to the Internet. While the caregivers are willing to share basic information via ICT in order to initiate contact with a volunteer, face-to-face contact with the potential volunteers of the respite service prior to beginning the job is important. Crucially, for this group of caregivers it is important that the volunteers belong to a volunteer service organization that takes responsibility for distributing human resources and follows up on the performance of the respite service provided.

The results suggest that the development of a peer-to-peer economic system is feasible, seeing that the key components of these systems was reflected in the discourse of the informal caregivers of dependent elders.

As a future work, we will evaluate potential volunteers’ perception on the use of ICTs to offer volunteer respite service for caregivers of dependent elders. Following with system design to provide connection between caregivers and volunteers.

## Appendix A. Questions that were used to guide the semi-structured interviews (translated from Spanish)

1. Have you previously received help from volunteers for the care of your family member? From who? What was your experience like?
2. Are you willing to receive help from a volunteer, to be with your family member so you can have some time off? Do you believe that your family member would be willing to have a volunteer take care of him/her for a period of time?
3. What type of help would you like to receive from a volunteer? How much time do you think the help should last? How often would you like to receive this help?
4. Which characteristics should the volunteer have?
5. What would help you trust a volunteer to take care of the elderly adult that you are in charge of?
6. If a computer-based system was available to help people communicate to give and receive this type of help, what do you imagine it would be like? How would the system work? Which information would you like to access? Which information are you willing to provide?
